# Carabid beetle diversity and mean individual biomass in beech forests of various ages

**DOI:** 10.3897/zookeys.100.1536

**Published:** 2011-05-20

**Authors:** Lucija Šerić Jelaska, Vlatka Dumbović, Mladen Kučinić

**Affiliations:** 1 Department of Zoology, Faculty of Science, University of Zagreb, Rooseveltov trg 6, 10000 Zagreb, Croatia; 2 State Institute for Nature Protection, Trg Mažuranića 5, HR-10100 Zagreb, Croatia

**Keywords:** carabid beetles, MIB, over-wintering stages, beech forests succession, Papuk Nature Park

## Abstract

Carabid beetle diversity and mean individual biomass (MIB) were analysed in three different successional stages of beech tree stands (60, 80 and 150 years old). Carabid beetles were captured using pitfall traps placed at nine sites (three per age class) in the Papuk Mountain of East Croatia during 2008. A cluster analysis identified three groupings that corresponded to the beech age classes. MIB values increased with stand age, ranging from 255 in 60-year-old stand to 537 in the oldest forests. The 80-year-old stand showed the highest species richness and diversity values. With respect to species composition, large species such as *Carabus scheidleri* and *Carabus coriaceus* were dominant only in the oldest forests. Furthermore, species that overwinter in the larval stage were more abundant in the oldest forests (45% of the total number of individuals from the 150-year-old stand) than in the younger ones (20% of individuals from 60-year-old, and 22% of individuals from 80-year-old stands). Our results showed that the analyses of species composition and life history traits are valuable for estimating the conservation values of older forests. Although the investigated sites form part of a continuous forested area and are only a couple of kilometres apart, MIB values detect significant differences associated with forest age and can be a useful tool in evaluating the degree to which a forest reflects a natural state.

## Introduction

In forest management, sustainability is an internationally accepted goal. One technique by which sustainability is assessed is through the monitoring of indicator species (Pearce and Venier 2006). Due to the large number of studies, particularly in the Northern Hemisphere, carabid beetles are one of the most frequently used biological indicators of boreal forest quality (Desender et al. 1994, Niemelä 2001, Niemelä et al. 2007). Several methods have been developed to quantify carabid beetle responses to environmental change and forestry practices (Dufrene and Legendre 1997, Koivula and Niemelä 2003, Rainio and Niemelä 2003).

Generally, it is assumed that older forests have less species richness than younger stages, but analysing species richness alone without considering species composition (e.g., forest specialists, saproxilic species) would underestimate the conservation value of older forests (Paquin 2008, Taboada et al. 2008). Succession is the change in community structure through time, where species characteristic of young stages are replaced with species characteristic of older stages (Schwerk and Szyszko 2007, Gotelli 2008). The end point of this process may be a climax community of insects that is invasion-resistant and cannot be replaced by other groups of insects until another disturbance event occurs (Gotelli 2008). For carabid beetles, such patterns have been observed during reforestation (after clear cutting or in plantations), where smaller carabids with better powers of dispersal were present in relatively greater abundance in younger forests than in older forests, which were dominated by larger, non-flying, and forest specialist species (Šustek 1981, Niemelä et al. 1993, Spence et al. 1996, Szyszko et al. 2000, Koivula 2002, Magura et al. 2002, Elek et al. 2005). In contrast to the wealth of studies on changes in carabid beetle diversity with boreal forest succession, little is known about this process in southern forests of Europe.

According to changes in species body sizes as well as their community composition, the Mean Individual Biomass (MIB) index of carabid beetles has been proposed as a good indicator of succession (Szyszko et al. 2000). Thus far, this index has been used to monitor succession in forest habitats (Serrano and Gallego 2004, Schwerk and Szyszko 2007) and post-industrial areas (Schwerk and Szyszko 2008) and to assess recovery after a mining accident (Cárdenas and Hidalgo 2007).

The aim of this study is to compare carabid beetle assemblages (species composition and richness) and MIB index values in mesophyllous beech forest stands of various successional ages (60-, 80- and 150-year-old forests). Our hypotheses are that the Mean Individual Biomass index will increase with forest age and species with over-wintering larvae will be more abundant in the older forests. Similar trends were evident from previous studies (Butterfield 1997, Magura et al. 2002) assessing carabid community succession in conifer plantations.

## Materials and methods

### Study area

**Figure 1.**
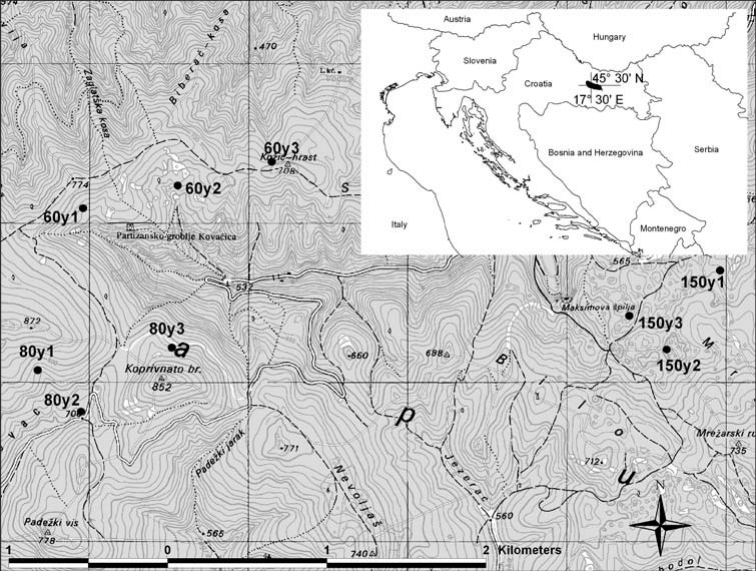
Position of investigated sites labelled according to the age of the forest (“60y 1–3” denote sites 1 to 3 in the 60-year-old forest, “80y 1–3” denote sites 1 to 3 in the 80-year-old forest and “150y 1–3” denote sites 1 to 3 in the 150-year-old forest). Insert: location of Papuk Nature Park in Croatia.

The study was performed in the Papuk Nature Park in continental Croatia (Fig. 1). The area was proclaimed protected in 1999 and represents a hilly and forested wildlife area within a lowland, agricultural region. The highest peak is 954 m above sea level (a.s.l.). The total area of Papuk Nature Park is 336 km2 and is mostly (96%) covered by forest, and the rest of the habitat consists of settlements and small agricultural areas. Sessile oak (*Quercus petraea* (Mattuschka) Liebl.) forests dominate areas up to 350 m a.s.l. Beech trees (*Fagus sylvatica* L.), depending upon geological substrate and microclimatic conditions, grow in several different forest associations and cover more than 50% of the forested area, whereas mixed beech-fir forests grow in areas higher than 700 m a.s.l. Forests are of natural origin but are influenced by forestry. Deciduous forests are managed as even-age stands. Beech-fir forests are managed following the selection cutting system. Logged areas are mainly under natural regeneration. Most forests in the Park are 60 to 80 years old. Jankovac forest (660 ha), which has been left unmanaged, is the only large beech-forest stand that is more than 150 years old. The carabid fauna was compared among three different beech forest stand ages: younger (60-year-old forest), middle-aged (80-year-old forest) and old beech forest (150 years old). For this study, we selected nine sampling sites (three per age class) (Fig. 1).

### Beetle sampling and data analyses

Carabid beetles were collected from mid May to mid September in 2008. We used plastic pitfall traps (0.5 L volume, 10 cm diameter) filled with a mixture of 96% ethanol, 9% acetic acid and water in equal proportions and covered with roofs for protection against rain and evaporation. At each sampling site, three individual traps were placed 10 m apart to form a triangle. Traps were emptied on the same day approximately every three weeks (a total of six visits).

Carabid beetles were collected using 27 traps from 162 trapping events altogether (3 traps x 9 sampling sites x 6 visits). Samples were pooled per site across all visits, resulting in 9 sampling sites (three per age class) for analyses.

The species collected were identified using keys (Hůrka 1996, Freude et al. 2004) and the Croatian Natural and History Museum collections in Zagreb. The total body length (from tip of abdomen to tip of mandibles) of each individual was measured (mm), and the mean body length of each species was calculated for all sampling sites. Species were also categorised according to their over-wintering strategy as adults or larvae (Thiele 1977, Hůrka 1996), and their frequency per sampling site was calculated.

To compare carabid diversity, we calculated Margalef species richness (number of species per standardised number of individuals), Shannon diversity index and Pielou’s evenness. For similarity between sites, Bray-Curtis indices using presence/absence data, and the number of individuals were calculated and used in cluster analyses with the complete linkage method for constructing dendrograms (Krebs 1989). Carabid beetle Mean Individual Biomass (MIB) was calculated to assess the stage of succession of the sites (Schwerk and Szyszko 2007, after Szyszko 1983). We used the following formula:

ln *y =* -8.92804283 + 2.5554921ln *x*,

where *x* is the body length of a specimen and *y* is the live estimated body weight of the individual. The estimated biomass of a species was calculated by multiplying the estimated body weight of the individual times its abundance for all sampled individuals per sampling site.

Differences in MIB values and diversity parameters among the three forest age classes were tested using analyses of covariance (ANCOVA), where mean individual biomass, species richness, evenness and Shannon diversity index were used as dependant variables, forest ages as categorical predictors and proportion of species with respect to overwintering strategy as the covariate, followed by the Scheffé post-hoc test. Pearson product-moment correlation coefficient was used for analyses. Statistical tests were performed using Primer 6 (PRIMER E Inc 2002) and Statistica 8 (Statsoft Inc 2008).

## Results

A total of 1244 carabid beetles belonging to 31 species were collected (Table 1). Between 9 and 18 species were collected per sampling site. Similar species richness levels have been recorded in other beech forests in nearby countries as well as in similar forest communities in Croatia (Elek et al. 2005, Magura et al. 2006, Šerić Jelaska and Durbešić 2009).

**Table 1.** List of species, their mean body sizes (authors’ measurements), over-wintering stage (a-adults, l-larvae), estimated body weight values and number of individuals in 60- , 80- and 150-year-old forests. Body weight values were calculated according to Szyszko (1983).

*Species*	*Mean body size /mm*	*Over-winteringstage*	*Body weight /mg*	*Number of individuals*		
				*60 y*	*80y*	*150y*
*Abax carinatus* (Duftschmid 1812)	16	a	158.4	139	228	133
*Abax parallelepipedus* (Piller & Mitterpacher 1783)	20	a	280.1	1	32	62
*Abax parallelus* (Duftschmid 1812)	17	a	184.9	0	26	15
*Aptinus bombarda* (Illiger 1800)	12	a	75.9	1	36	3
*Calosoma inquisitor* (Linné 1758)	21	l	317.3	2	1	1
*Carabus arcensis* Herbst 1784	20	a	280.1	36	38	1
*Carabus convexus* Fabricius 1775	17	a	184.9	1	0	1
*Carabus coriaceus* Linné 1758	39	l	1543.7	0	0	29
*Carabus intricatus* Linné 1761	30	a	789.5	3	0	1
*Carabus irregularis* Fabricius 1792	25	a	495.5	1	0	0
*Carabus nemoralis* O.F.Müller 1764	25	a	495.5	4	0	5
*Carabus scheidleri* Panzer 1799	30	l	789.5	0	16	119
*Carabus ullrichii* Germar 1824	25	a	495.5	3	53	20
*Carabus violaceus* Linné 1758	32	l	931.1	42	75	46
*Cychrus attenuatus* (Fabricius 1792)	17	l	184.9	0	6	1
*Cychrus semigranosus* Palliardi 1825	20	l	280.1	0	9	4
*Harpalus affinis* (Schrank 1781)	12	l	75.9	0	1	1
*Leistus piceus* Frölich 1799	8	a	26.9	0	1	1
*Licinus hoffmannseggii* (Panzer. 1797)	13	l	93.2	0	4	0
*Limodromus assimilis* (Paykull 1790)	11	a	60.8	0	1	0
*Molops elatus* (Fabricius 1801)	19	a	245.7	0	0	1
*Molops piceus* (Panzer 1793)	11	a	60.8	3	5	4
*Myas chalybeus* (Palliardi 1825)	16		158.4	4	1	0
*Notiophilus rufipes* Curtis 1829	6	a	12.9	0	0	1
*Platyderus rufus* (Duftschmid 1812)	7	l	19.2	1	0	0
*Platynus scrobiculatus* (Fabricius 1801)	11	l	60.8	0	1	0
*Pseudoophonus rufipes* (DeGeer 1774)	16	l	158.4	2	1	1
*Pterostichus aethiops* (Panzer 1796)	14		112.6	0	0	1
*Pterostichus niger* Schaller 1783	23	l	400.4	0	4	0
*Pterostichus oblongopunctatus* (Fabricius 1787)	12	a	75.9	0	8	0
*Pterostichus transversalis* (Duftschmid 1812)	16		158.4	0	3	0
*N (species)*				*15*	*22*	*22*
*N (individuals)*				*243*	*550*	*451*

The 80- and 150-year-old forests yielded 22 species each, whereas 15 species were collected from the 60-year-old forest sites. Nine species (29%) were present in all three forest age classes; one species was recorded only from the 60-year-old stand, six species only from the 80-year-old stand, and three only from the oldest forests.

The greatest number of individuals (44%) was recorded from the 80-year-old forest sites, 36% from the 150-year-old forest sites, and 20% from the 60-year-old forest sites (Table 1). Two species, *Abax carinatus* and *Carabus violaceus*,were collected from all nine sites with the greatest number of individuals,accounting for 40.2 and 13.1% of the total catch, respectively. Although *Abax carinatus* and *Carabus violaceus* were numerous at all three forest ages, two large species, *Carabus scheidleri* and *Carabus coriaceus*, were mainly collected from the 150-year-old sites. *Carabus scheidleri* and *Carabus coriaceus* accounted for 33% of the 451 individuals in the oldest forest and 12% of the total catch.

**Figure 2.**
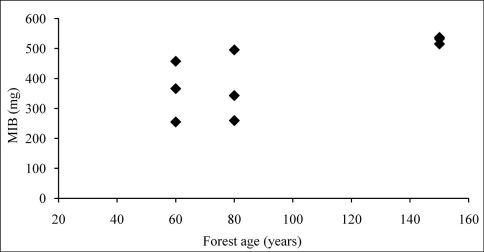
MIB values (mg) compared to the age of the forests stands (years).

**Figure 3.**
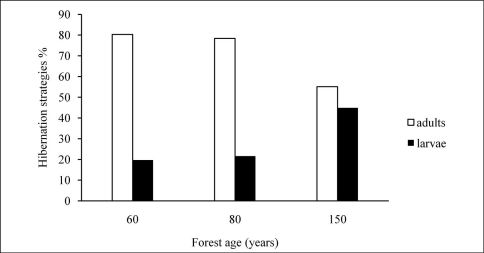
Proportion of species according to their hibernation strategies (larvae – black columns, adults – white columns) in relation to forest age (years).

The 80-year-old sites showed the highest average Shannon-Wiener index value, whereas the lowest value was recorded from the youngest sites. The variability of data in the whole sample could be explained by the second order polynomial with R2 = 0.54. The same trend was observed for the average standardised species richness (Margalef’s index) (Table 2). MIB values increased with forest age. Beech forests that are 150 years old have the highest mean individual biomass values for carabids (Table 2, Fig. 2). Similar trends were recorded for proportion of species that hibernate as larvae. There were more carabid beetles that hibernate as larvae (45% of individuals collected) in the oldest forest than in the younger forest stages; in the 60- and 80-year-old forests, 80 and 78% of specimens hibernate as adults, respectively (Fig. 3). MIB was highly correlated with the proportion of carabids that hibernate as larvae (r = 0.84, p < 0.05).

**Table 2.** Number of species and individuals, diversity indices and MIB (mg) values from the nine sites in Papuk Nature Park, Croatia.

*Plots*	*Total species*	*Total number of individuals*	*Margalef species richness*	*Pielou’s evenness*	*Shannon-Wiener index*	*MIB (mg)*
60y1	9	43	2.13	0.72	1.59	458
60y2	9	118	1.68	0.48	1.06	255
60y3	10	82	2.04	0.63	1.45	367
80y1	15	80	3.19	0.80	2.18	343
80y2	11	342	1.71	0.62	1.49	260
80y3	17	128	3.30	0.74	2.11	496
150y1	9	110	1.70	0.81	1.77	515
150y2	11	175	1.94	0.76	1.82	537
150y3	18	166	3.33	0.66	1.90	532
*Average values per age*						
60y	9.33	81.00	1.95	0.61	1.37	360
80y	14.33	183.33	2.74	0.72	1.92	366
150y	12.67	150.33	2.32	0.74	1.83	528

MIB values, Shannon–Wiener indices, Margalef’s indices and Pielou’s evenness did not differ significantly among forests of various age groups (ANCOVA, p<0.05), (Table 3). The covariate, hibernation strategy as larvae, was significantly related to the Mean Individual Biomass and Shannon–Wiener indices values (ANCOVA, p<0.05), (Table 3). Although they differed among forest age-groups at significance level slightly higher than p=0.05, performed post hoc comparison confirmed significant differences in MIB values of carabids between 150 year old forests and younger sites (60 and 80 year old forests) and for Shannon–Wiener indices between the 80-year-old sites from the youngest and the oldest sites (Scheffé test, p<0.05, Table 3).

**Figure 4.**
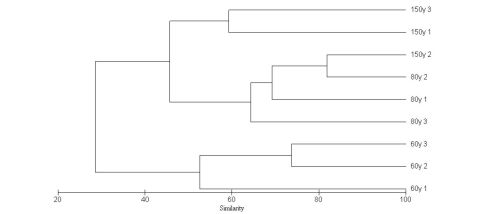
Dendrogram of cluster analyses among forest sites using presence/absence carabid beetle data. Two distinct clusters are formed at roughly 30% similarity. Marks 60y 1–3, 80y 1–3 and 150y. 1–3 denote investigated sites placed in the 60-, 80- and 150-year-old forests.

Based on presence/absence data, a cluster analysis identified three groups: one with all 60-year-old forest sites, one with all 80-year-old sites as well as one 150-year-old site, and a third group with two 150-year-old forest sites (Fig. 4). A cluster analysis based on the number of individuals, grouped 60-year-old sites with two 80-year-old sites and all 150-year-old sites with one 80-year-old site (figure not shown).

**Table 3.** ANCOVA results for the effects of forest age on mean individual biomass, the Shannon-Weiner diversity index, Pielou’s evenness and Margalef’s richness of carabid beetles, followed by the Scheffé post-hoc test.

*Variables*	*SS*	*DF*	*MS*	*F*	P	*Scheffé test*
*Dependant variable: MIB (g)*						
Corrected Model	0.100	3	0.033	39.883	0.001	
Intercept	0.079	1	0.079	95.071	0.000	
Species that hibernate as larvae	0.045	1	0.045	54.156	0.001	150>80=60
Forest age (years)	0.010	2	0.005	5.712	0.051	
Error	0.004	5	0.001			
R2 = 0.960 (Adjusted R2 = 0.963)						
*Dependant variable : Shannon-Wiener index*						
Corrected Model	0.796	3	0.265	7.268	0.028	
Intercept	2.184	1	2.184	59.831	0.001	
Species that hibernate as larvae	0.265	1	0.265	7.251	0.043	80>150=60
Forest age (years)	0.338	2	0.169	4.634	0.073	
Error	0.183	5	0.037			
R2 = 0.813 (Adjusted R2 = 0.702)						
*Dependant variable: Pielou’s evenness*						
Corrected Model	0.055	3	0.018	2.786	0.149	
Intercept	0.414	1	0.414	63.233	0.001	
Species that hibernate as larvae	0.026	1	0.026	3.948	0.104	
Forest age (years)	0.011	2	0.005	0.804	0.498	
Error	0.033	5	0.007			
R2 = 0.626 (Adjusted R2 = 0.401)						
*Dependant variable: Margalef richness*						
Corrected Model	1.651	3	0.550	1.099	0.431	
Intercept	3.558	1	3.558	7.106	0.045	
Species that hibernate as larvae	0.722	1	0.722	1.442	0.284	
Forest age (years)	0.919	2	0.459	0.918	0.458	
Error	2.504	5	0.501			
R2 = 0.397 (Adjusted R2 = 0.036)						

## Discussion

MIB values increased with forest age, confirming MIB as a useful indicator in evaluating later succession stages. An increase in MIB values was accompanied by changes in community structure, i.e., a decreasing portion of smaller carabids and an increasing proportion of the largest species, as well as their abundance, with forest age. A similar trend has been reported in Szyszko et al. (2000), Cárdenas and Hidalgo (2007), and (Schwerk and Szyszko (2007, 2008).

Although evenness slightly increased with forest age, other diversity parameters showed nonlinear patterns, with the highest average values for forests of mid-age. Cluster analyses of carabid composition in this study clearly divided the youngest and the oldest forests, whereas middle-aged forests occupied an intermediate position. Differences within 80-year-old forest sites were more pronounced than between sites from other age classes that show a more diverse data set. Similar results were found by Paquin (2008) who observed the highest variability in mid-aged classes during the natural regeneration cycle of burned forests.

Forest age could affect the carabids assemblages due to changes in habitat structure, where early and later forest successional stages differ in vegetation structure, and in the accumulation of dead and decaying wood (Tyrrell and Crow 1994, Ings and Hartley 1999). As older forests increase in resources and heterogeneity, they seem to support more large-sized carabid beetles. Due to habitat quality, mid-aged forests can be inhabited by species from younger and older forests, but because habitat resources might not be developed well enough to support the dominance of large species, overall carabid beetle biomass stay low despite showing greatest abundance. There are numerous studies focusing on carabid beetle body sizes among different habitats (Šustek 1987; Blake et al. 1994; Szyszko et al. 2000; Ribera et al. 2001; Braun et al. 2004; Weller and Ganzhorn 2004; Šerić Jelaska et al. 2007; Gaublomme et al. 2008; Šerić Jelaska and Durbešić 2009; Gómez 2010), with most of these confirming an increase in size with succession and habitat stability. Smaller carabid species develop faster with shorter generation times (Peters 1983, Blake et al. 1994), whereas larger carabid species have longer developmental periods (Blake et al. 1994) that can be supported in stable habitats with sufficient resources (Peters 1983, Lomolino 2005). As was shown here, younger beech forests were not characterised by large carabids, unlike the older forests. Carabid species that over-winter as larvae usually have larger adults as a result of a longer developmental period (Blake et al. 1994), which is supported by our results showing that the 150-year-old forests had a higher proportion of species that hibernate as larvae in comparison to the 80- and 60-year-old forests. Habitat conditions in the oldest forests without forest management practices are quite likely stable enough to support the dominance of large species and species with longer developmental periods. As such, these older forests are of considerable conservation value. The conservation value of these older forests is also supported by the study of bird communities. Mature forest specialists like the White-backed woodpecker (*Dendrocopus leucopterus*), which had previously been considered extinct in this part of Croatia, and the Red-breasted flycatcher, (*Muscicapa parva*) breeds only in the oldest forests in Papuk Nature Park (Dumbović 2007).

To summarise, MIB values showed significant differences associated with forest age and can be a useful tool in evaluating whether a forest reflects a natural succession. Furthermore, our study indicates that for preserving stable communities and overall carabid diversity, it is important to have part of the forest unmanaged or at least leave some stands to reach the decomposition phase, taking into account the spatial connectivity of stands enabling the migration of species.
